# Intrinsic TNF/TNFR2 Interactions Fine-Tune the CD8 T Cell Response to Respiratory Influenza Virus Infection in Mice

**DOI:** 10.1371/journal.pone.0068911

**Published:** 2013-07-09

**Authors:** Michael E. Wortzman, Gloria H. Y. Lin, Tania H. Watts

**Affiliations:** Department of Immunology, University of Toronto, Toronto, Ontario, Canada; McGill University, Canada

## Abstract

TNF is an important inflammatory mediator and a target for intervention. TNF is produced by many cell types and is involved in innate inflammation as well as adaptive immune responses. CD8 T cells produce TNF and can also respond to TNF. Deficiency of TNF or TNFR2 has been shown to affect anti-viral immunity. However, as the complete knockout of TNF or its receptors has effects on multiple cell types as well as on lymphoid architecture, it has been difficult to assess the role of TNF directly on T cells during viral infection. Here we have addressed this issue by analyzing the effect of CD8 T cell intrinsic TNF/TNFR2 interactions during respiratory influenza infection in mice, using an adoptive transfer model in which only the T cells lack TNF or TNFR2. During a mild influenza infection, the capacity of the responding CD8 T cells to produce TNF increases from day 6 through day 12, beyond the time of viral clearance. Although T cell intrinsic TNF is dispensable for initial expansion of CD8 T cells up to day 9 post infection, intrinsic TNF/TNFR2 interactions potentiate contraction of the CD8 T cell response in the lung between day 9 and 12 post infection. On the other hand, TNF or TNFR2-deficient CD8 T cells in the lung express lower levels of IFN-γ and CD107a per cell than their wild type counterparts. Comparison of TNF levels on the TNFR2 positive and negative T cells is consistent with TNF/TNFR2 interactions inducing feedback downregulation of TNF production by T cells, with greater effects in the lung compared to spleen. Thus CD8 T cell intrinsic TNF/TNFR2 interactions fine-tune the response to influenza virus in the lung by modestly enhancing effector functions, but at the same time potentiating the contraction of the CD8 T cell response post-viral clearance.

## Introduction

During an infection, the immune system must balance the need for a strong immune response against collateral damage. This is particularly true during respiratory infections where too strong a T cell response in the lung can cause immune pathology, but too weak a response can lead to failure to clear the infection, resulting in virus-mediated damage. Several members of the tumor necrosis factor receptor (TNFR) superfamily control the survival of T cells during viral infections [1–5]. TNF, the prototypical ligand of the TNFR family, binds two receptors, TNFR1 and 2, of which TNFR2 is the predominant receptor on CD8 T cells [6,7]. TNF exists in two forms, a membrane bound form (mTNF) and a soluble form (sTNF). TNF binds to both TNFR1 and TNFR2. Membrane TNF can trigger TNFR1 and TNFR2 signaling, whereas soluble TNF has preferential effects on TNFR1 over TNFR2 [8,9]. As millions of people are treated with TNF blocking agents to treat inflammatory diseases such as rheumatoid arthritis and Crohn’s disease [10], it is critical to understand the precise role of TNF in response to infection. Since the threat of new influenza pandemics is a constant and CD8 T cells are important in controlling influenza infection when neutralizing antibody responses are absent [11,12], the need to understand the impact of TNF signaling in influenza infection is particularly important.

The role of TNF in CD8 T cell responses appears to be context dependent. There is evidence that TNF binding to TNFR2 is costimulatory for T cells and can prolong the T cell response to *Listeria* or model antigens [13–16]. Moreover, TNF has been shown to be critical in enhancing the CD8 T cell response to weak tumor antigens, but is less important in the CD8 T cell response in a more robust acute viral infection model with lymphocytic choriomeningitis virus (LCMV) Armstrong [6]. On the other hand, the complete absence of TNF or its two receptors in mice has been shown to enhance the CD8 T cell response to viruses, such as LCMV and influenza virus [17–20].

As the complete absence of TNF leads to lymphoid architecture changes and affects many cell types [21–23], it has been difficult to assess the T cell intrinsic role of TNF in an immune response based on the above studies. TNF is produced by both lymphoid and non-lymphoid cells, including CD8 T cells, CD4 T cells, NK cells, macrophages, dendritic cells and epithelial cells and thus TNF could have indirect effects on CD8 T cell responses [24]. CD8 T cells produce TNF early upon antigenic stimulation [25,26] raising the question of the role of TNF intrinsically in the T cells when so many other cells can produce TNF. Others have used conditional knockout of TNF to examine the role of specific cellular sources of TNF in control of *Listeria monocytogenes* or *Mycobacterium tuberculosis*, as well as in organizing lymphoid tissues and in airway inflammation [27–30]. Here we ask specifically how TNF produced by T cells intrinsically influences the T cells. We use an adoptive transfer model in which TNF-deficient or TNFR2-deficient TCR transgenic T cells are transferred into an otherwise normal host, to test the effect of TNF or TNFR2 deficiency on the transferred T cells in the context of an otherwise normal immune system that can fully control the infection.

The results show that CD8 T cell intrinsic TNF/TNFR2 signaling has a dual role during influenza infection, in enhancing effector function per cell as measured by IFN-γ production and degranulation, as well as in potentiating the contraction of the effector response. This increased functionality but more rapid contraction of the CD8 T cell response may function to limit the damage that could be caused by a prolonged and inefficient effector response.

## Results

### Analysis of TNF and TNFR expression by CD8 T cells during influenza virus infection

**Figure 1:**
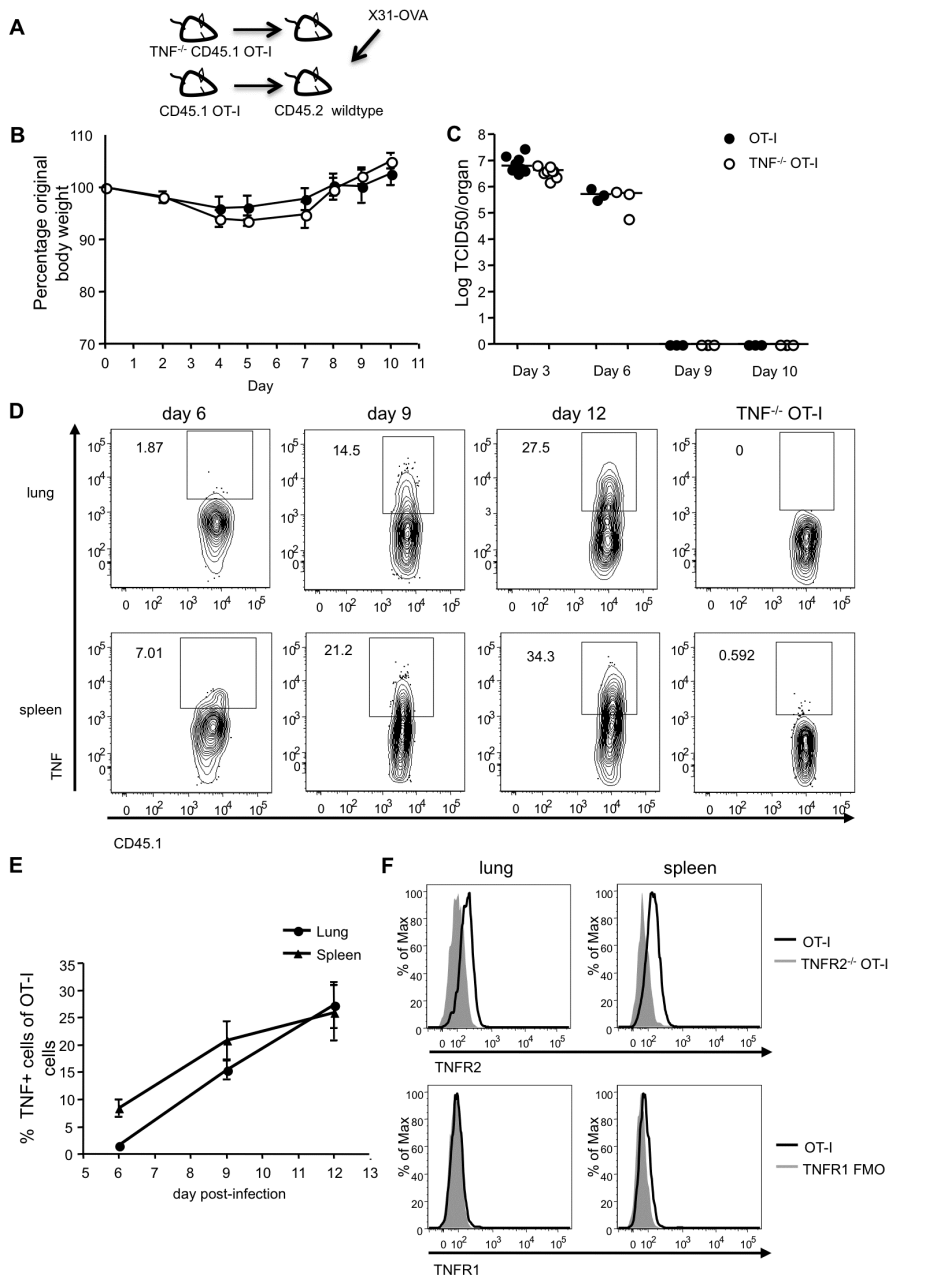
**Characterization of adoptive transfer model with respect to weight loss, viral clearance** and **TNF/TNFR expression.** (**A**) Ten thousand purified CD8 T cells from CD45.1^+^ OT-I or TNF^-/-^ CD45.1^+^ OT-I mice were injected intravenously into CD45.2^+^ wild type mice. A day later, the mice were infected with X31-OVA. (**B**) Infected recipients of either CD45.1^+^ OT-I or TNF^-/-^ CD45.1^+^ OT-I cells were monitored over time for weight loss, indicated as a percentage of original body weight. Data are representative of three independent experiments. (**C**) Lungs were excised from the animals at the indicated time points and analyzed for viral load as described in the methods. Data are representative of two independent experiments for day 3 and one for each day 6, 9 and 10, each using three to five mice per group. Day 3 and 6 or day 3 and 9 were done in the same experiment and results combined to show kinetics. (**D**, **E**) Cells from lung or spleen were restimulated with OVA_257-64_ peptide and Golgi Stop as described in the methods, surface stained for CD8 and CD45.1, fixed and then intracellularly stained for TNF, with representative FACS plots of TNF-positive cells, previously gated on CD8^+^ CD45.1^+^ cells (**D**) and the percentage of TNF^+^ OT-I cells summarized (**E**). For detailed gating strategy see Figure S1. Data are representative of two to four independent experiments for each time point, each using three to four mice per group. Data are pooled from experiments with day 6 and 9 or day 6 and 12 done in the same experiment. Error bars indicate SEM. (**F**) Representative histograms of TNFR2 and TNFR1 expression on lung and spleen OT-I cells day 7 post-infection. The dark line shows TNFR2 and TNFR1 on WT cells, and the shaded grey line represents TNFR2^-/-^ OT-I cells, or TNFR1 on the FMO sample. Data are representative of three independent experiments, each using three mice per group.

To assess the role of CD8 T cell intrinsic TNF in the response to influenza virus infection we crossed TNF^-/-^ mice with congenically marked CD45.1 OT-I TCR transgenic mice, whose TCR is specific for H-2K^b^ and OVA_257–264_ (SIINFEKL). This allowed us to monitor the effect of the absence of TNF on the transferred TCR transgenic T cells in a mouse in which all other cells express genetically normal levels of TNF. To analyze disease course in the mice, we transferred ten thousand purified naïve CD8 T cells from WT CD45.1 OT-I or TNF^-/-^ CD45.1 OT-I mice into separate CD45.2 WT mice and one day later infected the mice intranasally with influenza A/HK-X31-OVA (X31-OVA) (Figure 1A). This recombinant virus expresses the SIINFEKL epitope recognized by OT-I T cells in its neuraminidase stalk [31]. X31-OVA causes a mild infection associated with a reversible 5-10% weight loss and the virus is fully cleared by day 8 post-infection [11,32]. Mice that had received either WT or TNF^-/-^ OT-I T cells lost a similar amount of body weight and fully regained their original weight by day 8 (Figure 1B). Additionally, the kinetics of viral clearance in the lungs of TNF^-/-^ OT-I recipients was similar to those that received WT cells, such that both mice eliminated the virus between days 6 and 9 (Figure 1C), as previously reported for non-transgenic models of this infection [32]. As morbidity and viral load are similar between groups during this mild respiratory infection, this model is ideal to test the effect of T cell intrinsic TNF on CD8 T cells during the disease course, without the confounding effects of differential viral load, which can have profound impacts on T cell responses [33]. It should also be noted that in adoptive transfer models, the transfer of 5000 or more TCR transgenic naïve T cells substantially suppresses (>90%) the endogenous T cell response, such that the vast majority of the responding T cells in this model will be TNF deficient [34] and thus the endogenous T cells, which are all WT in this model, are unlikely to contribute substantially to the response.

To assess the potential role of intrinsic TNF in the T cell response, we first assessed the kinetics of TNF production by the WT OT-I T cells, using the TNF^-/-^ OT-I T cells as a staining control (Figure 1D). At day 6 post-infection, approximately 2% of the adoptively transferred OT-I T cells in the lung produced TNF upon restimulation, and this increased to 14.5% by day 9 and further increased to 27.5% by day 12 (Figure 1D, top panel). A similar pattern was observed for splenic OT-I cells (Figure 1D, bottom panel). The percentage of TNF-expressing OT-I cells recovered from both organs is summarized in Figure 1E.

To determine which of TNFs’ two receptors on the T cells could potentially respond to the intrinsically produced TNF, we next analyzed surface expression of TNFR1 and TNFR2 on the OT-I cells. TNFR2 has been previously shown to be the predominant TNF receptor on activated CD8 T cells, with minimal expression of TNFR1 [6,7], and this was confirmed upon examination of the OT-I T cells at day 7 post-influenza infection for both lung and spleen (Figure 1F). Similar results were seen on day 9 (data not shown). Thus TNFR2 is more predominantly expressed than TNFRI on the surface of the activated CD8 T cells during influenza infection in mice.

### CD8 T cell intrinsic TNF contributes to the contraction of the effector response to influenza virus in the lung

**Figure 2:**
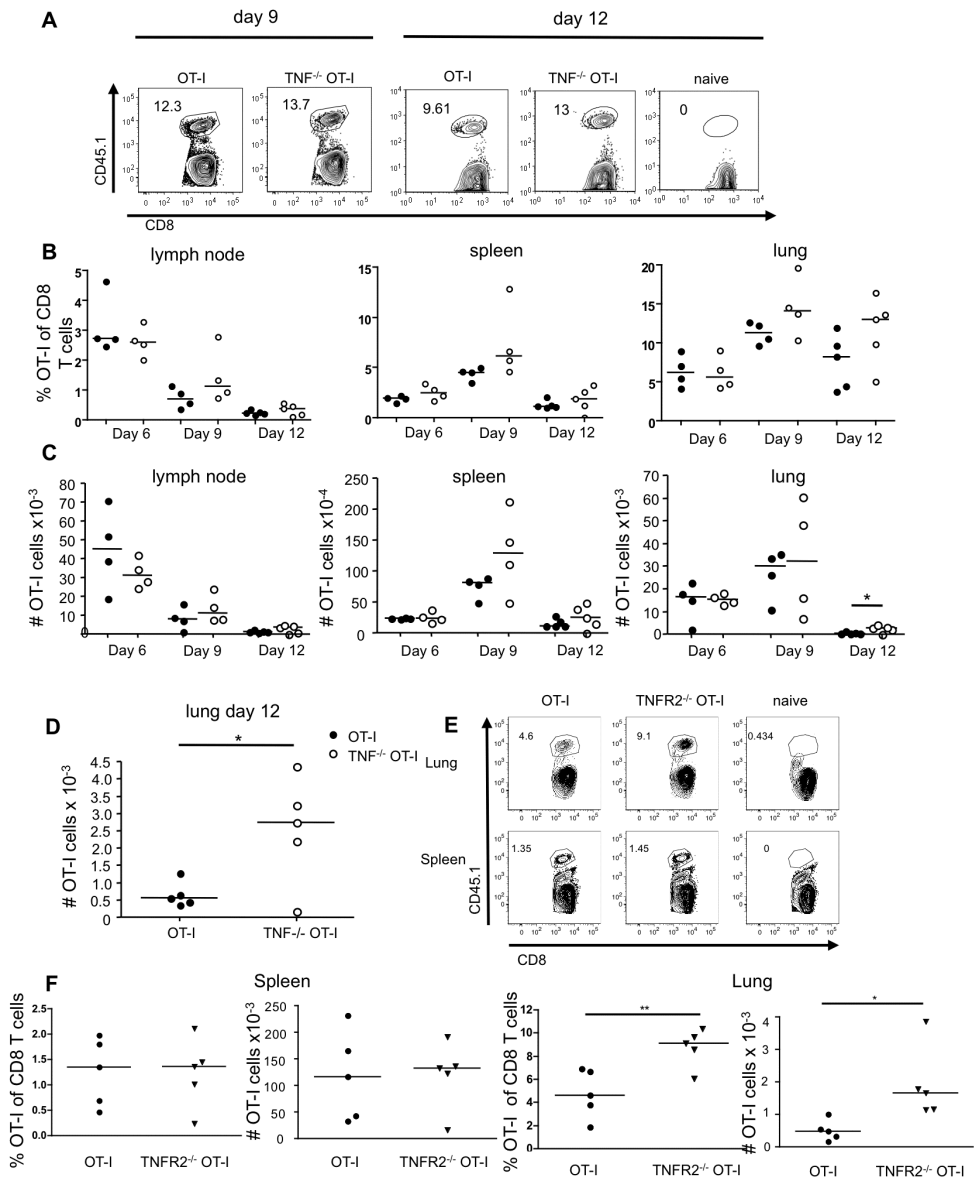
Intrinsic TNF/TNFR2 signals contribute to contraction of the CD8 T cell response during influenza infection. CD45.1^+^ OT-I or TNF^-/-^ CD45.1^+^ OT-I cells were transferred into wild type mice, and intranasally infected with X31-OVA one day later. Recovery of OT-I cells from the mediastinal lymph node, spleen and lung was determined. The naïve mouse did not receive transferred cells. (**A**) Representative gating of OT-I cells from the lung on day 9 and 12 post-infection. (**B**) Summary of the percentage of OT-I cells of CD8 T cells recovered at the indicated time points. (**C**) The absolute number of OT-I cells shown here was calculated by total organ cell count multiplied by the proportion of live, CD8^+^ CD45.1^+^ cells. (**D**) Day 12 lung data from panel C are shown on an expanded scale to highlight the difference in the number of OT-I cells. Data are representative of four independent experiments for day 6, two for day 9 and three for day 12, each using three to five mice per group. Data were collected with day 6 and 9 or day 9 and 12 done in the same experiment. (**E**, **F**) CD45.1^+^ OT-I and TNFR2^-/-^ CD45.1^+^ OT-I cells were transferred into wild type mice, followed by intranasal infection with X31-OVA one day later. Recovery of OT-I cells in the lung and spleen was determined on day 12. (**E**) Representative FACS plots of OT-I cells from the lung and spleen. (**F**) Summary of the percentage and absolute number of OT-I and TNFR2^-/-^ OT-I cells of CD8 T cells in the spleen (left) and lung (right). Data in E, F are representative of three independent experiments, each using three to five mice per group.

Having established that the OT-I T cells could express TNF and TNFR2 during an influenza-OVA infection, we next examined the impact of intrinsic TNF deficiency on the recovery of the OT-I T cells in the mouse from day 6 through 12 of infection (Figure 2A–D). Of note, the OT-I CD8 T cell numbers in the LN were highest at day 6, whereas the numbers of T cells increased in the lung between day 6 and 9, and then contracted again by day 12 (Figure 2C). WT and TNF^-/-^ OT-I T cells were recovered at similar numbers in the LN, spleen and lung at day 6-9 indicating that TNF^-/-^ OT-I T cells can expand as well as WT OT-I T cells (Figure 2B, C). On the other hand, by day 12 there were 2-5 fold more adoptively transferred (CD45.1) T cells in the lungs of mice that received TNF^-/-^ OT-I T cells as compared with mice that had received WT OT-I T cells (Figure 2C, and see amplified scale in Figure 2D). WT OT-I cells underwent ~40-fold contraction, compared to only ~13-fold for TNF^-/-^ OT-I cells. In sum, TNF^-/-^ OT-I T cells can undergo robust expansion leading to their accumulation in the lung, but show increased recovery in the lung at late time points, after virus has already been cleared from the lung. These data suggest a role for CD8 T cell intrinsic TNF in the contraction of the CD8 T cell response to influenza in the lung between days 9 and 12 post-infection. This loss of the WT CD8 T cells correlates with the timing of their highest capacity to produce TNF (Figure 1D,E).

### Intrinsic TNF mediates contraction of CD8 T cells through TNFR2

Although TNFR1 has been reported at low levels on CD8 T cells [6] and TNFR1 is involved in T cell proliferation in allogeneic models [35,36] we focused here on TNFR2/TNF interactions, as TNFR2 was the predominant receptor detected on the CD8 T cells in the influenza infected mice (Figure 1F). We hypothesized that CD8 T cell intrinsic TNF is acting in an autocrine fashion to mediate contraction through TNFR2. To test this hypothesis, we crossed TNFR2^-/-^ mice with CD45.1 OT-I mice. We infused WT CD45.1 or TNFR2^-/-^ CD45.1 OT-I cells into CD45.2 WT mice and analyzed their response to X31-OVA infection (Figure 2E, F). Similarly to the results observed with TNF^-/-^ OT-I cells, we observed a significantly greater proportion and absolute number of TNFR2^-/-^ OT-I cells compared to WT OT-I cells in the lung at day 12 post-infection (Figure 2E, F). In contrast, and as observed for the TNF^-/-^ OT-I T cells, we observed no difference in the recovery of WT or TNFR2^-/-^ OT-I T cells in the spleen.

To ask if TNFR/TNF interactions played a similar role in a secondary response, we transferred an equal number of in vitro generated memory-like OT-I CD8 T cells and infected with X31-OVA. Both WT and TNFR2^-/-^ OT-I memory CD8 T cells expanded and contracted to similar levels following influenza infection (data not shown). As memory CD8 T cells are more resistant to cell death, this may explain the TNF/TNFR2 independence of this secondary contraction [37].

### TNFR2-expressing CD8 T cells co-express intracellular TNF, but TNFR2 expression is associated with lower levels of TNF

**Figure 3:**
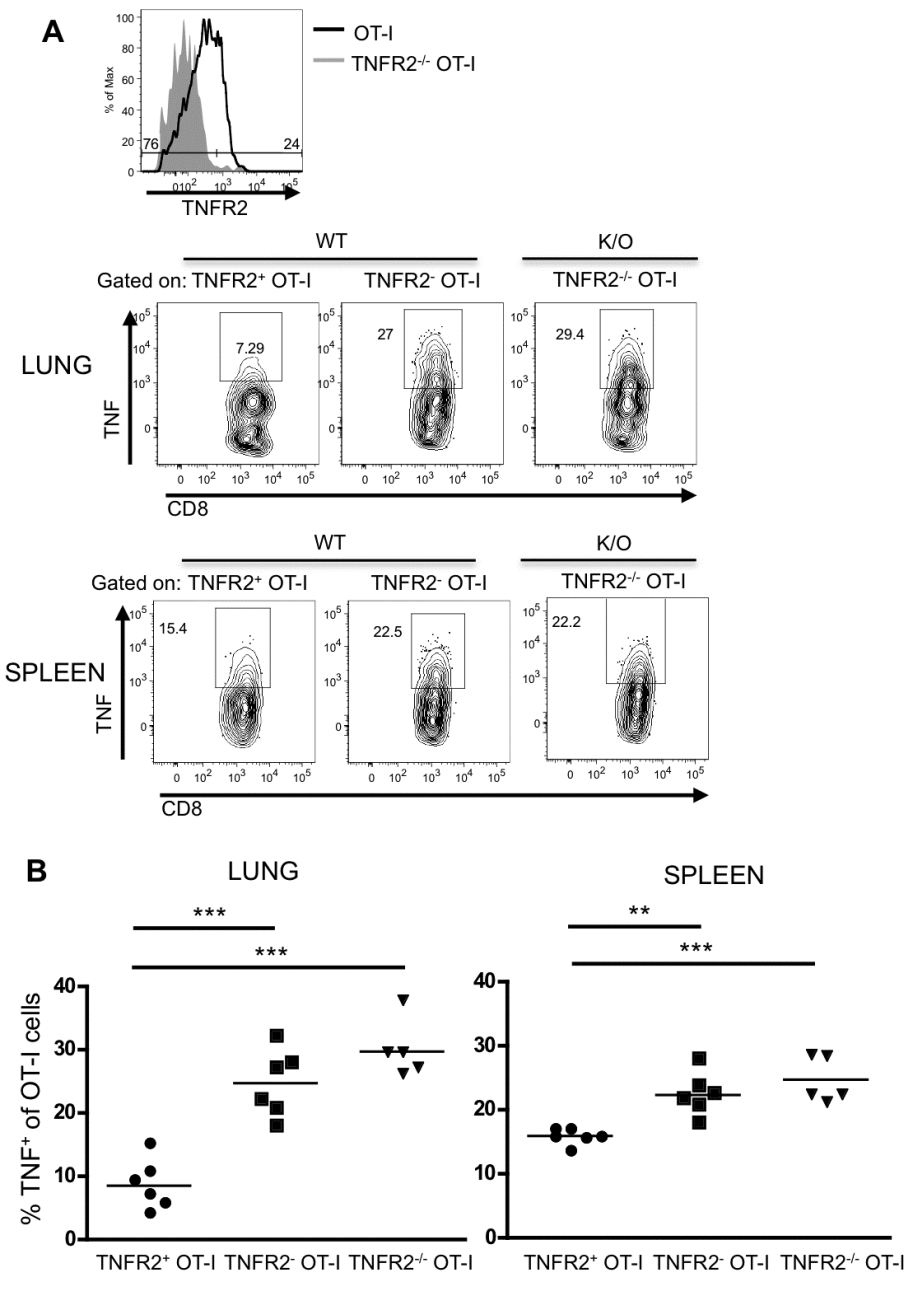
TNFR2 expression is associated with reduced TNF production. OT-I and TNFR2^-/-^ OT-I cells recovered from lung and spleen 10 days post-infection as in Figure 2 were restimulated at 37^0^C with OVA_257-64_ peptide and Golgi Stop for five to six hours. OT-I cells were then stained for surface TNFR2, followed by staining of intracellular TNF. (**A**) Representative FACS plot of TNFR2 expression on OT-I cells, using TNFR2^-/-^ OT-I cells as a control, and TNF production by WT TNFR2^+^, WT TNFR2^-^ and TNFR2^-/-^ OT-I subsets. (**B**) Summary of TNF production in lung and spleen. Each symbol represents one mouse.

Since TNF and TNFR2 in CD8 T cells play a role in their contraction following viral clearance, we investigated whether this could be due to an autocrine signaling mechanism. WT and TNFR2^-/-^ OT-I cells recovered from influenza-infected mice were restimulated with OVA peptide and stained for surface expression of TNFR2 followed by intracellular staining for TNF. Using TNFR2^-/-^ OT-I cells as a staining control, we found that a fraction of WT OT-I cells that express TNFR2 in the lung and spleen also produce TNF (Figure 3A, B). These results suggest that the same CD8 T cell subset that is producing TNF can also express TNFR2 on its surface, consistent with the possibility of autocrine signaling. Figure 3 also shows that TNFR2^-/-^ OT-I cells produce similar amounts of TNF as the WT OT-I cells that are negative for TNFR2 expression. Moreover, the amount of TNF detected in the TNFR2-negative WT and TNFR2^-/-^ OT-I cells is significantly more than the amount produced by the TNFR2^+^ WT OT-I cells (Figure 3A, B). Thus in primary T cells, TNF binding to TNFR2 may be inducing a negative feedback loop. Of interest, these effects are greater in the lung than in the spleen (Figure 3A, B).

### Phenotype of the CD8 effector T cells during influenza virus infection

**Figure 4:**
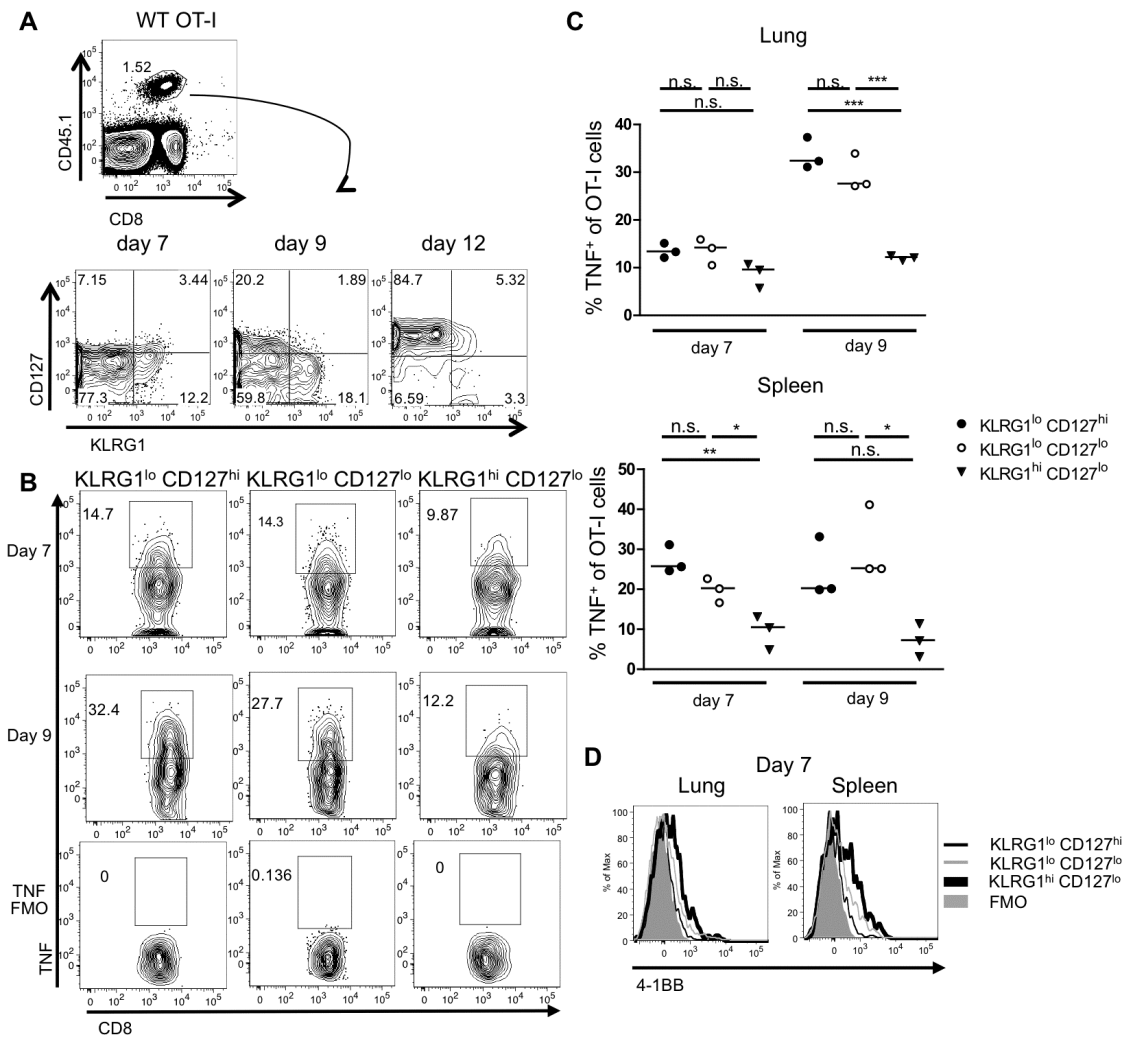
Characterization of OT-I effector cell subsets during influenza infection. (**A**) WT OT-I cells recovered from the lung and spleen of infected mice were stained for KLRG1 and CD127 expression. Representative FACS plots of KLRG1 versus CD127 on OT-I cells from infected spleens over time. (**B**) Restimulated OT-I cells from lung and spleen on day 7 and 9 were analyzed for TNF production. Representative FACS plots of TNF-positive OT-I subsets, gated on CD8^+^ CD45.1^+^ KLRG1^lo^ CD127^hi^, KLRG1^lo^ CD127^lo^ and KLRG1^hi^ CD127^lo^ cells from the lung. (**C**) Summary of TNF-positive OT-I subsets from lung and spleen on day 7 and 9. (**D**) Representative surface expression of 4-1BB on lung and spleen OT-I cells on day 7. The thick black line represents KLRG1^hi^ CD127^lo^, the thin black line represents KLRG1^lo^ CD127^hi^, the thin grey line represents KLRG1^lo^ CD127^lo^, and the shaded grey line represents the FMO control. Data from TNF-positive OT-I subsets are representative of two independent experiments. Data for 4-1BB levels are representative of three independent experiments, each using three mice per group.

We next investigated the phenotype of CD8 effector T cells that expand upon X31-OVA infection. Previous studies using LCMV infection of mice have identified that effector cells that are destined for death, termed short-lived effector cells, can be distinguished from memory precursor effector cells by their lower expression of CD127 and higher expression of inhibitory killer cell lectin-like receptor G1 (KLRG1) [38,39]. Thus, we stained the CD8 CD45.1 OT-I T cells for KLRG1 and CD127 following influenza infection of the mice (Figure 4A). This staining revealed three populations of OT-I effector T cells in the spleen and lung: KLRG1^lo^ CD127^hi^, KLRG1^hi^ CD127^lo^ and KLRG1^lo^ CD127^lo^ effector cells. Both KLRG1^lo^ CD127^hi^ and KLRG1^hi^ CD127^lo^ populations increased in size between day 7 and 9. The frequency of KLRG1^hi^ CD127^lo^ and KLRG1^lo^ CD127^lo^ effector cells drastically declined by day 12, whereas KLRG1^lo^ CD127^hi^ cells were the predominant population by this time point (Figure 4A).

A significantly greater proportion of KLRG1^lo^ CD127^hi^ cells had the capacity to produce TNF, compared to KLRG1^hi^ CD127^lo^ cells, with KLRG1^lo^ CD127^lo^ cells producing intermediate levels upon restimulation (Figure 4B, C). We also examined the three populations of OT-I effector cells for their levels of 4-1BB (Figure 4D). 4-1BB, whose expression is regulated by antigen receptor signaling on T cells [40], was most highly expressed on the KLRG1^hi^ CD127^lo^ cells in both the lung and spleen, with lower level expression on KLRG1^lo^ CD127^lo^ cells, and was undetectable on KLRG1^lo^ CD127^hi^ cells at day 7. By day 9, the expression of 4-1BB was undetectable on all the OT-I T cells (data not shown), consistent with previous results showing that 4-1BB expression is related to recent antigen stimulation [40,41] and lost after viral clearance [3]. TNFR2 and GITR expression were similar between subsets on day 7 and 9, whereas TNFR1 was undetectable (data not shown). Taken together, based on 4-1BB expression, it is likely that the KLRG1^hi^ CD127^lo^ cells have received the highest level of antigen stimulation, whereas the KLRG1^lo^ CD127^hi^ cells may have seen less antigen. Upon restimulation the KLRG1^hi^ cells produce significantly less TNF than the other effector subsets, consistent with their being more terminally differentiated [38].

### Phenotype of TNFR2^-/-^ CD8 T cells

**Figure 5:**
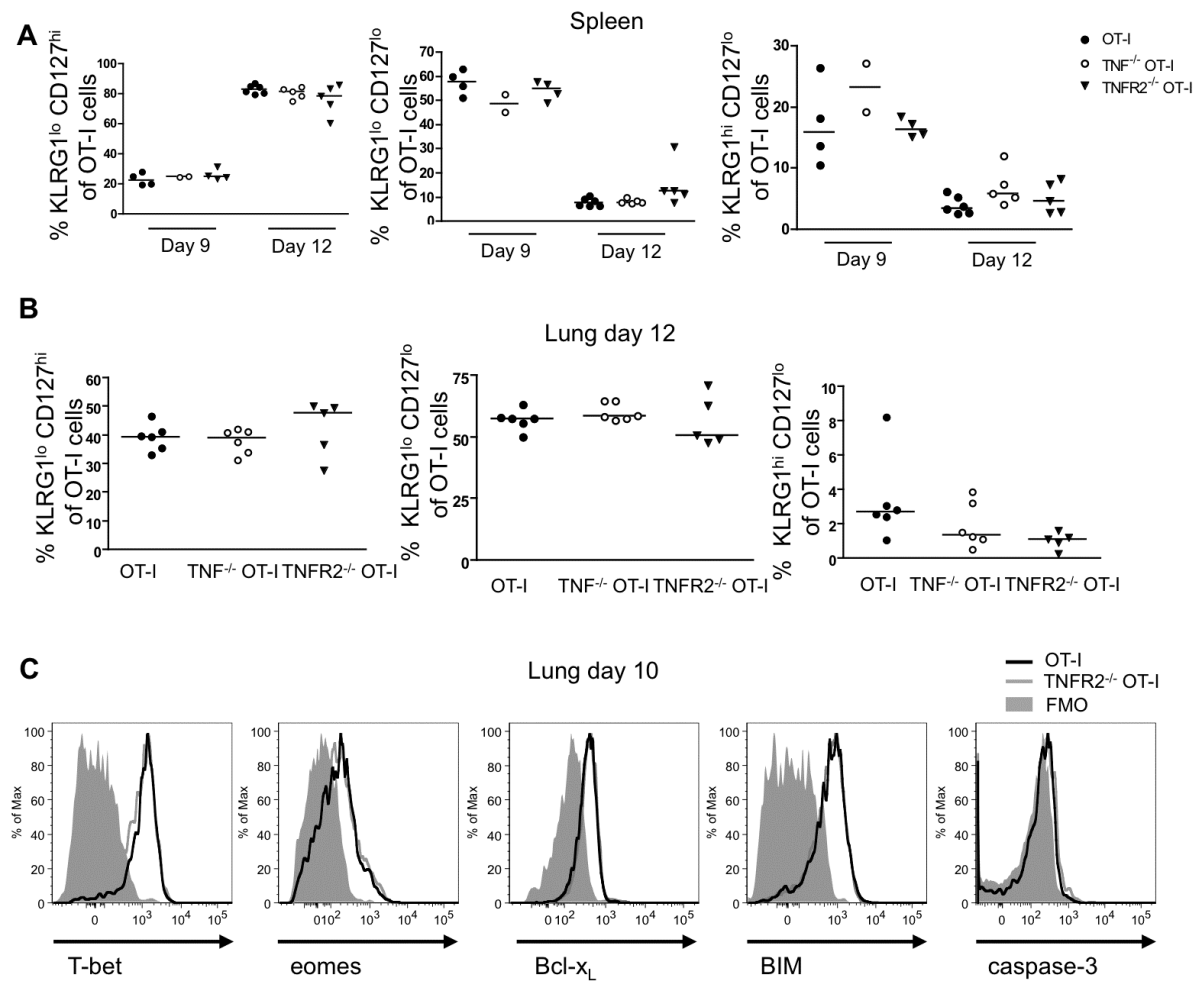
Characterization of effects of TNF on contraction and transcription factor expression of/by CD8 T effector subsets. (**A**)Summary of percentage of OT-I subsets based on KLRG1 and CD127 expression from mice that received OT-I, TNF^-/-^ OT-I or TNFR2^-/-^ OT-I cells recovered from the spleen on day 9 and 12. (**B**) Percentage of OT-I subsets from the lung on day 12. Data are representative of three independent experiments, each using two to six mice per group. (**C**) Direct ex vivo expression of intracellular T-bet, eomes, Bcl-x_L_, BIM and caspase-3 on OT-I and TNFR2^-/-^ OT-I cells recovered from the lung on day 10. Data are representative of two independent experiments, each using four to six mice per group.

To determine the effect of TNF/TNFR2-mediated contraction on the three different effector populations, we stained WT, TNF^-/-^ and TNFR2^-/-^ CD45.1 OT-I T cells for KLRG1 and CD127 expression in the lung and spleen (Figure 5). The proportions of KLRG1^lo^ CD127^hi^, KLRG1^lo^ CD127^lo^ and KLRG1^hi^ CD127^lo^ cells in both TNF^-/-^ and TNFR2^-/-^ OT-I cells were similar to WT OT-I cells in the spleen between day 9 and 12 (Figure 5A) and in the lung on day 12 (Figure 5B). Thus intrinsic TNF/TNFR2 mediated contraction in the lung does not discriminate between the CD8 effector subsets defined by KLRG1/CD127 staining. To determine whether differential transcription factor expression or an imbalance of intracellular pro-survival and pro-death molecules could explain the enhanced accumulation of TNFR2^-/-^ OT-I cells or function, we analyzed the expression of T-bet, Eomes, Bcl-x_L_, BIM, and caspase-3 on WT and TNFR2^-/-^ OT-I cells isolated on day 10. Expression of these molecules was similar between WT and TNFR2^-/-^ OT-I cells. Thus differences in survival molecules between WT and TNFR2^-/-^ are not apparent, perhaps reflecting the rapid clearance of dead and dying cells in vivo.

### CD8 T cell intrinsic TNF/TNFR2 enhances cytokine production and degranulation

**Figure 6:**
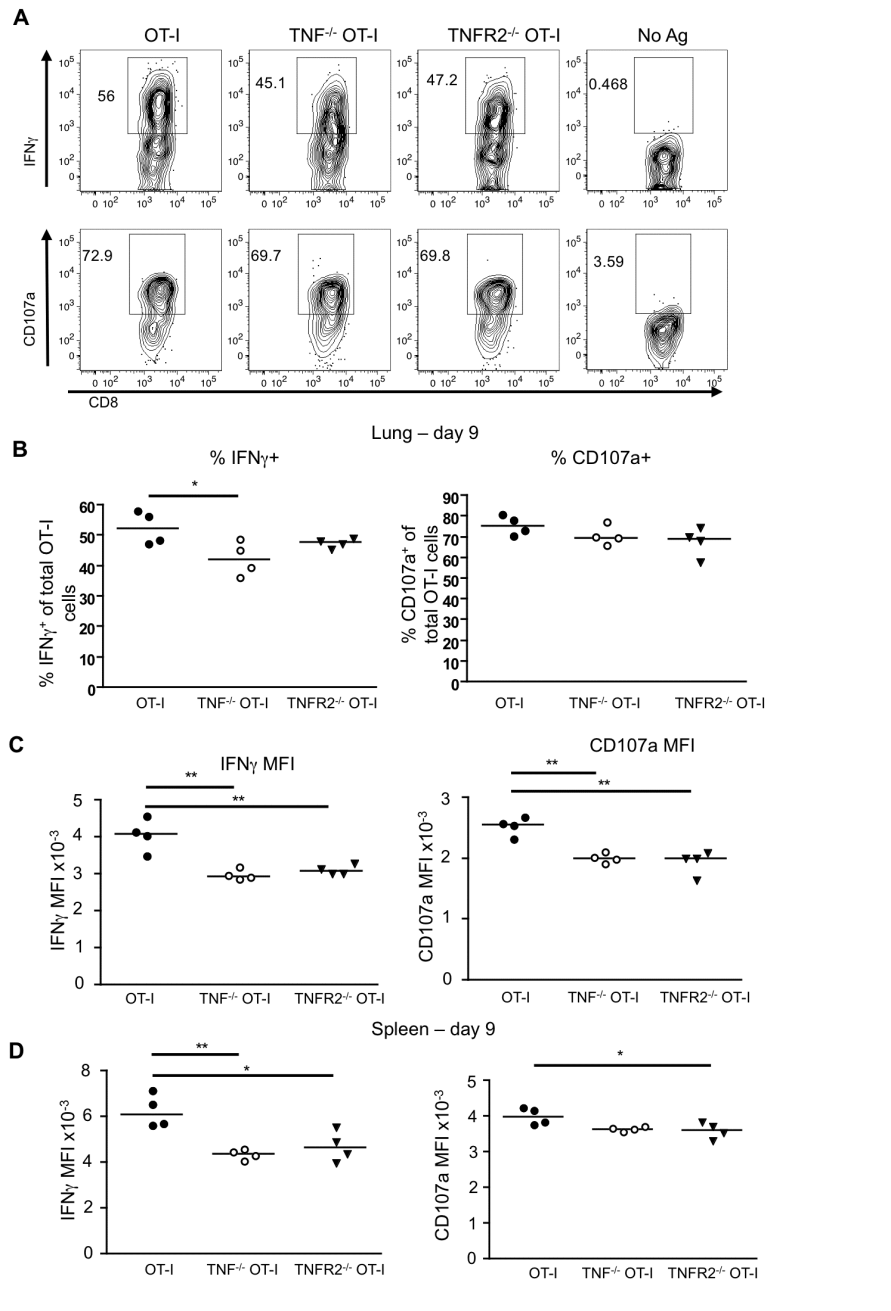
CD8 T cell intrinsic TNF enhances both IFN-γ production and degranulation. WT, TNF^-/-^ and TNFR2^-/-^ OT-I cells recovered from infected lungs and spleens as in Figure 2, were restimulated at 37^0^C with OVA_257-64_ peptide and Golgi Stop for five to six hours. Cells were then analyzed for production of IFN-γ and expression of CD107a. (**A**) Representative FACS plots of IFN-γ and CD107a staining from day 9 post-infection in the lung. Cells that were not restimulated (No Antigen) as well as FMO staining were used as controls. (**B**) Percentage of OT-I cells that are positive for IFN-γ and CD107a on day 9 in the lung. (**C**) MFI of IFN-γ and CD107a for IFN-γ- and CD107a-positive OT-I cells, respectively. (**D**) MFI of IFN-γ and CD107a from spleen samples on day 9. Data are representative of five independent experiments for day 9-12 for TNF^-/-^ OT-I cells, and three for TNFR2^-/-^ OT-I cells, each using four mice per group.

We next asked whether intrinsic TNF/TNFR2 activity was modulating not only the quantity of the CD8 T cells, but also their functionality. Lung and spleen cell suspensions from infected WT, TNF^-/-^ or TNFR2^-/-^ OT-I cell recipients were restimulated with SIINFEKL peptide and the expression of intracellular IFN-γ, as well as CD107a, a marker of degranulation, were determined (Figure 6). The proportion of TNF^-/-^ OT-I cells expressing IFN-γ was marginally reduced compared to WT OT-I cells, but this was not recapitulated with the TNFR2^-/-^ cells (Figure 6A, B). However, the MFI of IFN-γ and CD107a was significantly decreased in the absence of intrinsic TNF or TNFR2 in both the lung (Figure 6C) and the spleen (Figure 6D). Similar results were seen on day 12 (data not shown). This defect was only observed during the peak and contraction phase of the response, as lung-resident TNF^-/-^ OT-I cells were indistinguishable from WT OT-I T cells on day 6 post-infection (data not shown). Thus intrinsic TNF/TNFR2 interactions increase the level of IFN-γ production per cell as well as the extent of degranulation by lung CD8 T cells.

## Discussion

TNF has been extensively characterized as an important mediator in several inflammatory disorders, but until now, its CD8 T cell intrinsic role during influenza infection has been unclear. In this study we utilized an adoptive transfer model with TNF^-/-^ and TNFR2^-/-^ OT-I CD8 T cells to investigate this issue. In this model, only the transferred T cells lack TNF or TNFR2 in the context of an otherwise completely normal immune system. Importantly, our model allowed us to ask about the intrinsic effects of TNF and TNFR2 on T cells under conditions where viral load was not different between mice receiving WT, TNF or TNFR2 deficient T cells. Interestingly, during the response to influenza virus, the CD8 T cells showed increased capacity for TNF production over time, continuing until very late in the response, after virus had been cleared. CD8 T cell-derived TNF appears to enhance effector function of CD8 T cells as evidenced by increased IFN-γ and CD107a MFI on the WT T cells compared to TNF^-/-^ and TNFR2^-/-^ T cells upon restimulation, and at the same time increases the contraction of the CD8 T cell response after viral clearance. The finding that largely similar effects were obtained with TNF^-/-^ and TNFR2^-/-^ OT-I T cells suggests that TNF is acting in an autocrine fashion by binding to TNFR2 on CD8 T cells. Consistent with this, a fraction of the CD8 T cells were found to co-express both TNFR2 and TNF. Thus we have identified a dual role for autocrine signaling by TNF/TNFR2 in CD8 T cells, in enhancing CD8 T cell functionality, but also in increasing their contraction through TNF binding to TNFR2. It is of interest that TNFR2 is preferentially triggered by mTNF, whereas sTNF has little impact on TNFR2 signaling [9]. Thus mTNF must act locally, binding to TNFR2 on the same cell or an adjacent T cell, which is consistent with the autocrine/paracrine signaling we have identified here.

The results presented here show that TNF contributes to the contraction of the CD8 T cell response in the lung, implying a role for TNF/ TNFR2 interactions in promoting cell death, while paradoxically enhancing the level of effector function per cell. These opposing roles of TNFR2 in promoting death of the effectors while promoting IFN-γ production and degranulation are likely explained by TNFR2 inducing degradation of the downstream signaling molecule TRAF2. Engagement of TNFR2 by TNF on T cells has been shown to result in the cIAP-1 dependent degradation of TRAF2, potentiating TNF mediated apoptosis [42]. Moreover, this loss of TRAF2 induced by TNFR2, but not by TNFRI signaling, renders primary T cells more sensitive to activation induced cell death [43]. TRAF2 is an upstream activator of classical NF-κB and thus loss of TRAF2 prevents classical NF-κB pathway dependent prosurvival signaling by other TNFR family members [43]. Indeed, the TRAF2 dependent TNFRs 4-1BB and GITR have been shown to prolong CD8 T cell survival in the lung during influenza infection, thus TNFR2-dependent loss of TRAF2 may contribute to a decrease in survival signaling through these receptors [3,4]. While these events have been well established in vitro [42,43], the lack of good flow cytometry antibodies for TRAF2 has made it difficult to test this hypothesis on cells immediately ex vivo. In support of a role for TNFR2 in promoting T cell death, Kim et al. found that memory CD8 T cells lacking TNFR2 underwent greater expansion than WT TCR transgenic T cells and offered superior tumor control, attributed to resistance of the TNFR2^-/-^ CD8 T cells to activation induced cell death [44].

The loss of TRAF2 induced by TNFR2 likely also explains the enhanced production of IFNγ by TNF^-/-^ or TNFR2^-/-^ cells. Activation of the alternative NF-κB pathway is normally restricted in resting cells by a complex between TRAF2/3/cIAP1/2, which act as an E3 ligase complex to degrade the upstream activator NIK [45,46]. In primary T cells, the TNFR2 dependent degradation of TRAF2 results in activation of the alternative NF-κB pathway [9]. Moreover, activation of the alternative NF-κB pathway in T cells leads to increased production of IFNγ and TNF [47].

The effect of autocrine TNF on contraction of the CD8 T cell response was restricted to the lung compared to the spleen and lymph nodes. This may reflect that effector cell contraction in the spleen and LN reflects migration of effectors to the tissues, whereas in the lung, the T cells persist until they become terminally differentiated. It was recently shown using overexpression of human TNFR2 in a TNFR1/2 deficient mouse embryonic fibroblast line that TNFR2 is internalized with its ligand, leading the authors to speculate that TNFR2 internalization provides a negative feedback loop [48]. Consistent with that hypothesis, we observed greater T cell derived TNF production in the absence of TNFR2, and more so in the lung compared to the spleen (Figure 3), suggesting that TNF levels may be higher in the lung, thereby contributing to TNF effects on contraction in that organ.

TNF and TNFR2 have been shown to contribute to the contraction of T cell responses in other contexts. For example, TNFR2 deficiency in all cells was found to contribute to the contraction of the CD8 response to LCMV, but only when combined with TNFR1 deficiency [19]. Turner et al. [20] found enhanced CD8 T cell responses to influenza in mice lacking TNFR2 on all cells after secondary infection, but not during the primary response. In the Turner et al. study the additional absence of TNFR2 on other cell types may have masked the role for TNFR2 in contraction of the primary CD8 T cell response to influenza seen in the present study. In contrast, in the present study, memory T cells appeared to be resistant to effects of TNF on CD8 T cell contraction (data not shown). In another recent study, the absence of TNF in all cells was found to result in increased inflammation, greater weight loss and increased numbers of macrophages, polymorphonuclear cells and antigen-specific T cells in the airway lumen during influenza infection, consistent with our T cell results [18]. In the study of Damjanovic et al. [18] the absence of TNF from all cells may have resulted in greater inflammation, resulting in increased weight loss, which we did not see in our study, perhaps because in our study TNF deficiency was limited to the adoptively transferred T cells.

Our study examined the T cell intrinsic role of TNF using an adoptive transfer model in which only the transferred T cells lack TNF. This has the advantage of allowing us to assess the specific role of TNF/TNFR2 in the T cell response, in the context of an otherwise normal host. One disadvantage of this adoptive transfer model is that the T cells that we use have developed in the absence of TNF in other cells and thus one cannot completely rule out an effect of TNF during T cell development. Another feature of the adoptive transfer model is that it suppresses the endogenous T cell response [34], such that we are focusing on an immune response to one immunodominant high affinity epitope. An advantage of the adoptive transfer model is that a small number of purified WT and gene deficient T cells are transferred into genetically and environmentally identical hosts, alleviating concerns that the gene knockout induces unexpected global changes in the host, such as for example, microbiome alteration.

Others have used conditional knockout approaches to assess the effects of cell specific deletion of TNF [27–30]. In the case of infection with *Listeria monocytogenes*, macrophages and neutrophils were found to be the main source of TNF after bacterial challenge, and this source of TNF was critical to mouse survival, with smaller effects of T cell produced TNF on survival of mice to *Listeria* challenge [28]. Interestingly, T cell intrinsic TNF is critical for mouse survival at later stages of *M. tuberculosis* infection [27]. These studies [27,28] have largely focused on the cellular source of TNF acting extrinsically to control bacterial load, in contrast to the present study, where our specific question was on how TNF produced by T cells acts on the T cells themselves.

In contrast to the results shown here, several studies have shown that TNFR2 provides a costimulatory signal to T cells, contributing to their clonal expansion, in vitro and in vivo [13–15]. Similarly, T cell intrinsic TNF was shown to promote T cell survival in a TCR transgenic model involving peptide stimulation [49]. Thus in several viral infection models, as well as in secondary responses to tumors it appears that TNFR2 is contributing to CD8 T cell contraction [16,18–20,44]; whereas with model antigens as well as during primary *Listeria* infection [13–15], TNFR2 appears to play a prosurvival role. TNFR2 has in common with 4-1BB the recruitment of TRAF1 and TRAF2 [50–52] TRAF2 provides a key link between TNFRs and NF-κB induced survival signaling [53]. On the other hand, engagement of TNFR2 on T cells has been shown to promote cIAP1 dependent TRAF2 degradation [42], which in turn would limit survival signaling downstream of TNFR1, as well as other TRAF2-binding TNFRs. Thus the role of TNFR2 in pro-survival or pro-death signaling may depend on the timing and extent of TRAF2 degradation during TNFR2 signaling. This context dependent role of TNFR2 in costimulation versus contraction of the T cell response may depend on factors such as the level and duration of TNF signaling in the particular model and/or the state of differentiation and level of survival molecules in the CD8 effectors at the time they receive their TNF signal.

Evidence in the literature suggests that the contraction of the CD8 T cell response is pre-programmed early after infection and takes place independently of pathogen clearance [54]. Previous reports have identified that IFN-γ directs the contraction phase of the CD8 T cell response to *Listeria* infection [55]. In the present study, we observed a major contraction of the CD8 T cell response between day 9 and 12 independently of T cell intrinsic TNF/TNFR2, however, the presence of TNF/TNFR2 on the T cells augments this contraction phase. The lack of TNF/TNFR2 signaling results in an additional 1000-1500 antigen-specific CD8 T cells on day 12 post-infection compared with mice that received WT cells. Although small, this number is likely biologically significant, in that the precursor frequency of T cells specific for a given antigen in a mouse ranges from 5–200 [56]. Moreover, transfer of as few as 1000 OT-I effector T cells can protect from an otherwise lethal influenza infection [3]. Thus we suggest that while not the major mechanism of T cell contraction following influenza infection, T cell intrinsic TNF fine-tunes the final stages of contraction of the response after viral clearance. The combined effects of feedback regulation of TNF production by TNFR2 signaling on CD8 T cells as well as the role of T cell intrinsic TNF in potentiating contraction of the CD8 T cell response could prevent damage from prolonged T cell activation after viral clearance.

A previous study showed that the antigen-inducible TNFR 4-1BB can prolong CD8 T cell effector responses in the lung when virus persists, but that 4-1BB is rapidly downregulated when virus is cleared, thus allowing more rapid contraction of the response [3]. Of interest, like TNFR2 [57], 4-1BB recruits TRAF1 and TRAF2 [50,52]. However, 4-1BB uses TRAF1 and TRAF2 for prosurvival signaling through the classical NF-κB pathway [47,50]. In contrast, TNFR2 signaling induces TRAF2 degradation to induce the alternate NF-κB pathway [9] and here we have shown that CD8 intrinsic TNF/TNFR2 signaling potentiates contraction of the CD8 T cell response in the lung after viral clearance. These differential effects might be explained by the mode of interaction of the TRAF2 protein with the TNFR cytoplasmic tails influencing whether they are a substrate for cIAP-mediated K48-linked ubiquitination and degradation or K63-linked ubiquitination to induce classical NF-κB activation and survival [58–60]. It is of interest that in mild influenza infection, 4-1BB is no longer detected on the lung T cells by day 9, under conditions where TNFR2 is still present (data not shown). Thus TNFR2 may be potentiating CD8 T cell contraction after 4-1BB is no longer providing survival signals to the T cells. These results ( [3] and this report) highlight the differential effects of 4-1BB and TNFR2 in fine-tuning immunity to influenza virus in the lung. We suggest that the effect of TNF/TNFR2 interactions in enhancing IFNγ and degranulation by CD8 T cells and later inducing increased contraction of the response, offers a way of ensuring precise control of the CD8 T cell response to influenza virus.

## Materials and Methods

### Mice

Male C57BL/6 (B6) mice were obtained from Charles River Laboratories (Wilmington, MA). TNF^-/-^ (B6.129S6-*Tnftm1Gkl*/J) or TNFR2^-/-^ (B6.129S2-*Tnfrsf1btm1Mwm*/J) both backcrossed for 10 generations onto C57BL/6 background, were obtained from the Jackson Laboratory (Bar Harbor Maine). The TNF^-/-^ and TNFR2^-/-^ mice were crossed with CD45.1 OT-I TCR transgenic mice (generated in our laboratory by crossing OT-I and CD45.1 mice obtained from The Jackson Laboratory, Bar Harbor, ME) to generate TNF^-/-^ CD45.1 OT-I and TNFR2^-/-^ CD45.1 OT-I mice. Mice were housed under specific pathogen-free conditions in sterile microisolator cages.

### Ethics statement

Mouse studies were approved by the University of Toronto animal care committee in accordance with the regulations of the Canadian Council on Animal Care, approved protocol number 20009458.

### T cell isolation and adoptive transfers

CD8 T cells were purified from the spleens of naive male CD45.1 OT-I, TNF^-/-^ CD45.1 OT-I or TNFR2^-/-^ CD45.1 OT-I mice using a negative selection mouse CD8 T cell enrichment kit (StemCell Technologies, Vancouver, Canada). The purified CD8 T cells were injected intravenously (i.v.) at 10^4^ cells/mouse in 200µl into 6-8 week old male B6 mice. One day later, mice were infected with X31-OVA, as described below.

### Influenza virus infection

B6 mice that had received CD45.1 OT-I, TNF^-/-^ CD45.1 OT-I or TNFR2^-/-^ CD45.1 OT-I T cells were infected with 1.25 hemagglutinin units (HAU) influenza A/HK-X31-OVA (X31-OVA) [31], provided by P. Doherty and P. Thomas (St. Jude Children’s Research Hospital, Memphis, TN). At the indicated times post X31-OVA infection, spleens, mediastinal lymph nodes and lungs were harvested. Single-cell suspensions were prepared from the specified organs and stained for flow cytometry. The lungs were first perfused with PBS, and lymphocytes were enriched by isolation over an 80/40% Percoll gradient.

### Viral Clearance

Lungs were excised from mice at various time points after X31-OVA infection and then homogenized in RPMI 1640 medium (1g lung tissue/10ml). Supernatant was obtained and stored at -70°C. TCID(50) was determined by the MDCK assay with the REED and Muench technique as previously described [61].

### Flow Cytometry

Adoptively transferred OT-I cells were surface stained with fluorescently-tagged antibodies against the following: CD8α (BD Biosciences, San Jose, CA and eBioscience, San Diego, CA), CD45.1 (BioLegend, San Diego, CA), KLRG1, CD127, GITR, secondary streptavidin, eomesodermin (eomes), T-bet (eBioscience, San Diego, CA), Biotin-TNFR1, Biotin-TNFR2, caspase-3 (BD Biosciences, San Jose, CA), Bim (Alexis Biochemicals, New York), Bcl-x_L_ (Cell Signaling Technology, Beverly, MA), biotin-4-1BB, and in some cases fixable viability dye (eBioscience, San Diego, CA). For intracellular TNF and IFN-γ staining, splenocytes and lymphocytes isolated from lungs were restimulated at 37°C with 1µM OVA_257-64_ using Golgi Stop (BD Biosciences, San Jose, CA) for 6 and 5 h, respectively. Cells were then surface stained for CD8 and CD45.1, fixed, and then intracellularly stained for IFN-γ and TNF (BD BioSciences). Five µg/milliliter anti-CD107a (BD Biosciences) was added at the beginning of the restimulation culture for detection of degranulation. Fluorescence minus one (FMO) controls and unstimulated samples (no peptide added) were used as negative controls. Following staining, samples were analyzed using a FACSCalibur or FACSCanto (BD Biosciences) and FlowJo software (Tree Star, Ashland, OR).

### Statistical Analysis

Each symbol is representative of one mouse per group. Where indicated for comparison of two values, p values were obtained using the Student’s *t* test (unpaired, two tailed, 95% confidence interval). One-way ANOVA was used to compare multiple samples. Statistically significant differences between groups are indicated as *p < 0.05, **p < 0.01, or ***p < 0.001.

## Supporting Information

Figure S1 Gating strategy for tracking OT-I cells in vivo. Transferred CD45.1 OT-I cells from infected CD45.2 mice were recovered as follows: cell suspensions isolated from lung, spleen and lymph node were gated on live cells with FSC-A x SSC-A, singlets with SSC-W vs SSC-H, negative staining of fixable viability dye, CD8+ cells, followed by CD45.1+ cells. Representative gating of a spleen sample from a WT CD45.1 OT-I recipient mouse, using an uninfected mouse that did not receive OT-I cells as a negative control.
(TIF)
