# Supplementary figures and images for: Intrinsic TNF/TNFR2 Interactions Fine-Tune the CD8 T Cell Response to Respiratory Influenza Virus Infection in Mice
Source: PLoS One. 2013 Jul 9;8(7):e68911. doi: 10.1371/journal.pone.0068911 (PMC3706430; doi:10.1371/journal.pone.0068911)

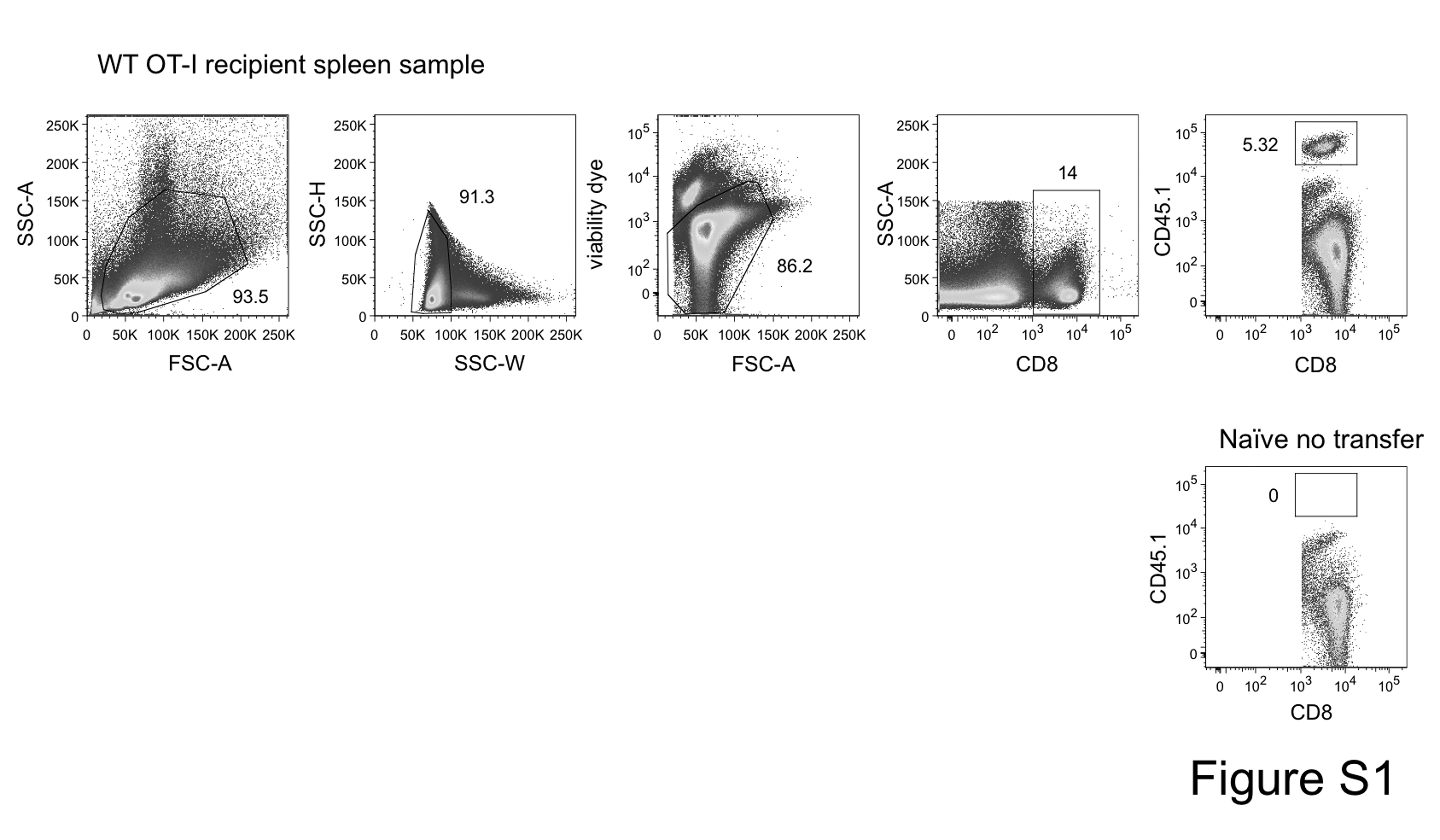

Supplement: Figure S1 — Transferred CD45.1 OT-I cells from infected CD45.2 mice were recovered as follows: cell suspensions isolated from lung, spleen and lymph node were gated on live cells with FSC-A x SSC-A, singlets with SSC-W vs SSC-H, negative staining of fixable viability dye, CD8+ cells, followed by CD45.1+ cells. Representative gating of a spleen sample from a WT CD45.1 OT-I recipient mouse, using an uninfected mouse that did not receive OT-I cells as a negative control. (TIF) [file pone.0068911.s001.tif]
